# Comparison of PD‐L1 detection assays and corresponding significance in evaluation of diffuse large B‐cell lymphoma

**DOI:** 10.1002/cam4.2316

**Published:** 2019-05-31

**Authors:** Sixia Huang, Lin Nong, Li Liang, Yalin Zheng, Wei Wang, Jumei Liu, Dong Li, Xin Li, Ying Wang, Bo Zhang, Ting Li

**Affiliations:** ^1^ Department of Pathology Peking University First Hospital Beijing China; ^2^ Department of Pathology Peking University Health Science Center Beijing China

**Keywords:** DLBCL, FISH, IHC, PMBCL, RNAscope

## Abstract

The expression of programmed cell death ligand 1 (PD‐L1) is a biomarker for immunotherapy, but approved detection method is absent in diffuse large B‐cell lymphoma (DLBCL). Here, we performed three methods including immunohistochemistry (IHC) (clone SP263 and SP142), RNAscope, and fluorescence in situ hybridization (FISH) to evaluate PD‐L1 status on a cohort of DLBCL including 94 of DLBCL‐NOS, 25 of primary mediastinal large B‐cell lymphoma (PMBCL) and 7 of double‐hit lymphoma (DHL). SP263 with 25% for immune cell (IC) or combined cell and SP142 with 10% for tumor cell (TC), 20% for both of IC and combined cell were proved to have corresponding survival prognostic. Combined^+^ showed comparable prognostic value with TC^+^ and IC^+^. SP263 and SP142 showed strong concordance (*k* = 0.788) with combined^+^ rates of 33.3% (42/126) and 34.9% (44/126), respectively. In DLBCL‐NOS, TC^+^ by SP263 preferred to non‐GCB and immunoblastic variant DLBCL‐NOS (*P* = 0.029 and *P* = 0.004). Combined^+^ (SP263 and SP142) were associated with more than one extranodal site involved (*P* = 0.006, *P* = 0.042), higher ECOG PS scores (*P* = 0.001, *P* < 0.001), high IPI risk (*P* = 0.012, *P* = 0.005), and poor treatment response (*P* = 0.095, *P* = 0.002). IC^+^ by SP263 and SP142 were both independent risk factors (*P* = 0.027, *P* = 0.037). 9p24.1 locus amplification and gain were identified in 4.3% and 7.6% DLBCL‐NOS and indicated shorter overall survival (*P* = 0.004). Positive rate of PD‐L1 by RNAscope was 36.5%, while no clinical significance shown. PD‐L1 positive rates were all higher in PMBCL and DHL than in DLBCL‐NOS by SP263, SP142, RNAscope, and FISH (*P* = 0.001, *P* < 0.001, *P* = 0.005 and *P* < 0.001, respectively). In conclusion, combined PD‐L1 expression by IHC was potentially reliable and convenient as a predicting biomarker. SP263 staining was easier to evaluate and recognized more PD‐L1‐stained cells, but SP142 presented a better prognostic indicator. FISH and RNAscope could be used as supplementary assays. PMBCL itself was a sensitive cohort for immunotherapy.

## 1 INTRODUCTION

Diffuse large B‐cell lymphoma (DLBCL) is the most common subtype of non‐Hodgkin lymphoma in adults, accounting for approximately 25%‐35% of all newly diagnosed non‐Hodgkin lymphomas in developed countries, and the proportion is even higher in developing countries.^1^ DLBCL consists of a group of highly heterogeneous tumors. Over the past decades, the rapid expansion of biotechnologies helped us understand the genetic basis of heterogeneity of DLBCL.^2^ Gene expression profiles (GEP) have identified at least three distinct molecular subtypes according to a cell‐of‐origin classification: germinal center B‐cell‐like (GCB), activated B‐cell‐like (ABC), and primary mediastinal B‐cell lymphoma (PMBCL), and approximately 20% of cases remain unclassified.^3^, ^4^

The standard first‐line treatment of DLBCL relies on CHOP‐like cytotoxic chemotherapy combined with immunotherapy using rituximab (anti‐CD20), and it has improved outcomes in DLBCL, where approximately 65% of patients achieve prolonged complete remissions (CR); however, 30%‐40% of cases still develop into relapsed/refractory (R/R) disease.^5^ Recently, various targeted therapies and newer agents that have emerged in DLBCL include immune checkpoint inhibitors, which promote the cytotoxic activity of tumor‐specific T cells by blocking the delivery of inhibitory signals conveyed by tumor cells to immune cells and leading to stimulation of the antitumor immunity mediated by T cells. One of the most promising approaches is antibody blockade of the programmed cell death‐1 (PD‐1)/ programmed cell death ligand‐1 (PD‐L1) pathway, which has shown significant clinical activity in both solid tumors and hematologic malignancies.^6^, ^7^

Not all patients respond to PD‐1/PD‐L1 blockade. Currently, the main biomarkers for the selection of patient cohorts include PD‐L1 overexpression, microsatellite instability (MSI), and high tumor mutational burden (TMB).^8^, ^9^, ^10^ Among them, detection of PD‐L1 expression is the most common and convenient method. Currently, five PD‐1/PD‐L1 blockade agents have been approved by the US Food and Drug Administration (FDA) for patients after the failure of first‐line therapy in several solid tumors and classical Hodgkin lymphoma, and a “one drug, one assay” immunohistochemistry (IHC)‐based method with four corresponding diagnostic primary antibodies (22C3, SP263, SP142, and 28‐8) was also approved for PD‐L1 detection.^11^ PD‐L1 expression on tumor cell (TC) and tumor‐infiltrating mononuclear immune cells (IC) were both measured, and the cutoff was variously based on different antibody clones.^12^ In DLBCL, the consensual diagnostic method has not been approved by the FDA as yet, but several ongoing clinical trials are investigating the use of drugs in DLBCL.^13^ Most studies used IHC method, but the expression level of PD‐L1 was not consistent owing to these assays being dependent on the specific kind of antibodies, cutoff values, companies, and companion instruments used.^14^, ^15^, ^16^ Recently, other methods were also considered used as a predictive marker. Previous studies suggested that patients with 9p24.1/PD‐L1/PD‐L2 amplification could also respond to PD‐1/PD‐L1 blockade, which was more obvious in tumors with higher incidences of 9p24.1 amplification, such as in primary mediastinal large B‐cell lymphoma (PMBCL).^17^, ^18^ In addition, antibody‐independent methods such as RNA‐seq or in situ hybridization (ISH) have been used to measure the mRNA level of PD‐L1 in solid tumors, revealing a high association with PD‐L1 immunostaining and even higher sensitivity.^19^, ^20^ However, the role of 9p24.1 amplification and PD‐L1 mRNA levels in the diagnostic assay of PD‐L1 in DLBCL is still unknown, and a more broadly applicable diagnostic method is needed.

In the described study, the protein expression of PD‐L1 was detected by IHC with the antibodies SP263 and SP142, the mRNA level was measured by RNAscope, and the PD‐L1 genetic locus alteration of 9p24.1 was analyzed by fluorescence in situ hybridization (FISH) in a cohort of DLBCL, including DLBCL‐NOS, PMBCL, and double‐hit lymphoma (DHL). The status of PD‐L1 from different molecular levels was determined by the three methods and was compared. At the same time, the corresponding prognostic roles of PD‐L1 detected by each of the methods were analyzed. The investigation aimed to identify a more practical and reliable method for PD‐L1 detection for potentially predicting the response of DLBCL to PD‐1/PD‐L1 inhibitors in clinical practice.

## 2 MATERIALS AND METHODS

### 2.1 Case selection

One hundred and twenty‐six cases of large B‐cell lymphomas were selected from the files of the Department of Pathology, Peking University First Hospital. Among the 126 cases, 101 cases of DLBCL‐NOS were obtained from 2011 to 2015, while 25 cases with PMBCL were obtained from 2008 to 2017. All the 126 cases were reclassified according to the 2017 World Health Organization (WHO) classification of tumors of hematopoietic and lymphoid tissues. DLBCL‐NOS was defined as cases with diffuse proliferation of neoplastic large B cells but that could not be classified as any other specific subtype of DLBCL, and EBV‐positive cases were also excluded. As a new entity, cases with high‐grade B‐cell lymphoma, with rearrangements of MYC and BCL2 and/or BCL6, which was also called “double‐hit” or “triple‐hit” lymphoma (THL), were selected out from cases initially diagnosed as DLBCL‐NOS. PMBCL was confirmed based on the site of involvement (mediastinum), morphology (diffuse infiltration of large cells and fibrotic background), and distinctive immunophenotype (expression of CD20, CD30, and/or CD23). Six cases of reactive hyperplasia lymph nodes were selected as the control group. All 126 cases had adequate specimens of formalin‐fixed paraffin‐embedded (FFPE) samples, of which 30 DLBCL‐NOS, 9 PMBCL, and 3 DHL were embedded into six TMA blocks with a manual tissue arrayer (Quick‐Ray Manual Tissue Microarrayer, Unitma, Korea). The clinical information of the patients was obtained by reviewing the medical digital database; 117 of the 126 cases had available clinical data, and 110 of the 126 cases had complete follow‐up data.

### 2.2 Immunohistochemistry (IHC)

To assay the PD‐L1 protein expression level, two antibodies (clone SP142; clone SP263) were used for the 126 cases. IHC with SP263 was performed on freshly cut 4‐μm‐thick FFPE tissue sections using the BenchMark ULTRA automated staining platform (Ventana, Tucson, AZ, USA) with an Optiview DAB IHC Detection Kit according to the manufacturer's instructions. Every section stained with the PD‐L1 rabbit monoclonal primary antibody (clone SP263, Ventana) was set up with a matched rabbit immunoglobulin G negative control and a positive control from the placenta. IHC of SP142 was carried out with PD‐L1/PAX5 immunohistochemical double staining according to a previous procedure.^16^ Briefly, after blocking with 0.3% hydrogen peroxide for 10 minutes, the 4‐um‐thick slides were incubated with the anti‐PAX5 monoclonal mouse antibody (clone EP156, Zhongshan, Beijing, China) at 4°C overnight. The slides were stained with alkaline phosphatase (AP) horse anti‐mouse IgG (ZB‐2310, 1:50, Jackson Immunoresearch, PA, USA) for 30 minutes and incubated with the AP‐Red detection kit (ZLI‐9042, Zhongshan, Beijing, China) for 15 minutes for visualization of PAX‐5 (red). The slides were then incubated with the anti‐PD‐L1 rabbit monoclonal antibody (clone SP142, 1:100, Spring Bioscience, San Francisco, USA) at 4°C overnight, followed by peroxidase‐conjugated AffiniPure goat anti‐rabbit IgG (ZB2301, 1:100, Jackson ImmunoResearch, PA, USA) incubation for 30 minutes and diaminobenzidine staining for 10 minutes to visualized PD‐L1 (brown).

**Figure 1:**
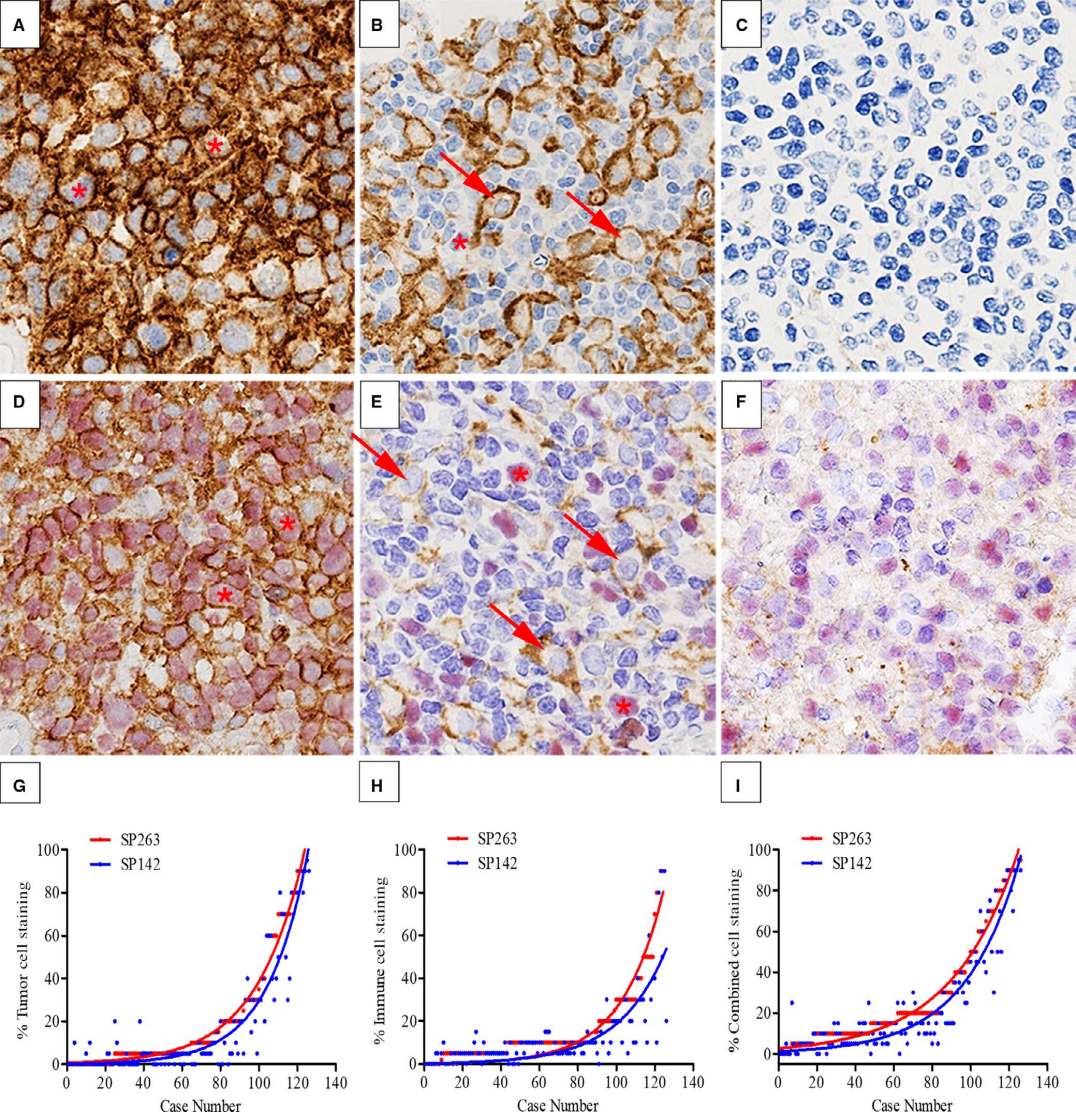
Overexpression of programmed cell death ligand‐1 (PD‐L1) at the protein level in diffuse large B‐cell lymphoma (DLBCL). A‐C (×400) SP263 staining. Tumor cells (TC) and immune cells (IC) showed strong membranous staining. The yellow stars indicated positive TC, and the red arrows indicated positive IC (A, B). Negative samples with no staining (C); D‐F (×400) SP142 staining. TC^+^ DLBCL showed double staining of tumor cells with PD‐L1 (brown) and the B‐cell specific marker PAX‐5 (red) (represented by yellow stars) (D). IC^+^ DLBCL showed staining with only PD‐L1 highlighting macrophages (represented by red arrows) (E). The negative samples showed no brown staining (F); G‐I The comparison of percentage positive tumor cells, immune cells, and combined cells staining, by case, for SP263 and SP142. Each point represents the mean percentage score of PD‐L1 expression cells from two pathologists for each assay on each case

The scoring algorithm of PD‐L1 expression in our study was performed as reported.^16^, ^21^ For TC, only partially or completely membranous staining was considered as PD‐L1 TC^+^, and TPS (number of positive TCs/ number of all TCs) was estimated; for ICs, predominantly macrophages and lymphocytes (Figure 1), membranous and/or cytoplasmic staining were both considered as PD‐L1 IC^+^, and the percentage of positive ICs/ total tumor tissue cellularity was estimated. Recently, combined positive score (CPS) was approved by FDA in the utilized of 22C3 antibody in gastric and gastroesophageal junction adenocarcinoma.^22^ Different from solid tumor, it was hard to distinguish TC from IC in DLBCL. For easier to assess, we tried a modified CPS method to evaluate the PD‐L1 combined^+^ (percentage of positive TCs and ICs/ total tumor tissue cellularity) in DLBCL. The percentage of PD‐L1 staining cells in our study was mainly scored by two experienced hematopathologists, and the mean percentage of positive cells was used for analysis. The disputed cases were confirmed by a third hematopathologist. The determination of positive cutoff value for SP263, SP142, and the detailed reasons is described in the Results section.

The antibodies used for classified cell‐of‐origin (COO) and double‐expressor lymphoma (DEL) were anti‐CD10 (clone 56C6, 1:50, Zhongshan), anti‐BCL6 (clone PG‐B6p, 1:40, Dako), anti‐MUM1 (clone MUM1p, 1:50, Dako), anti‐BCL2 (clone 124, 1:100, Dako), anti‐c‐Myc (clone EP121, 1:75, Zhongshan). Immunohistochemical staining was performed on 10% formalin‐fixed, deparaffinized 4‐um sections using routine methods. Color development was carried out using 3,3′‐diaminobenzidine tetrahydrochloride. We use a cutoff of 50% with BCL‐2 expression and 40% with c‐Myc expression to define DEL.^23^

### 2.3 Fluorescence in situ hybridization (FISH)

FISH was performed on 126 DLBCL FFPE samples according to the manufacturer's instructions. PD‐L1 (9p24.1/CD274) Dual‐Color probe (F.01244, Anbiping, Guangzhou, China) was performed as previously reported.^24^ Samples with two red signals targeting PD‐L1 and two green signals targeting ABL1 were classified as normal. The presence of three or four red signals was determined as gain, whereas 5 red signals or more were classified as amplification. The probe signals for a monolayer of at least 200 tumor cell nuclei were counted per sample at 100x magnification, and alterations were called when exceeding a 20% threshold in the number of nuclei.^24^

MYC (8q24) break apart rearrangement probe (F.01054; Anbiping, China); BCL2/IGH fusion translocation t (14; 18) probe (F.01066, Anbiping, China) and BCL6 (3q27) break apart rearrangement probe (F.01069, Anbiping, China) were performed and evaluated according to the manufacturer's instructions, and the threshold all was 20%.

### 2.4 RNAscope

PD‐L1 mRNA levels by ISH with RNAscope were detected in 58 samples, including 30 cases of DLBCL‐NOS, 25 cases of PMBCL, and 3 cases of DHL (six TMA samples and the remaining 16 samples were PMBCL FFPE puncture cases). An RNAscope assay (Advanced Cell Diagnostics, ACD, Hayward, CA) with the probes Hs‐PAX5‐C1 (469981, ACD) and Hs‐CD274‐C2 (600861‐C2, ACD), positive control probes Hs‐PPIB‐C1/POLR2A‐C2 (321641, ACD), negative control probe DapB (320751, ACD), and RNAscope® 2.5 HD Duplex Reagent kit (cat# 322430, ACD) was performed following the manufacturer's instructions. In brief, 5‐μm‐thick paraffin‐embedded TMA sections were baked for 60 minutes, deparaffinized, and then boiled with the pretreatment reagent for 15 minutes. Protease digestion was carried out at 40°C for 30 minutes, followed by probe hybridization for 2 hours at 40°C with target probes against PD‐L1 and PAX5. Hybridized signals were amplified and visualized with the RNAscope 2.5 HD Duplex Reagent kit. PD‐L1‐positive signals showed as red punctate dots in the cytoplasm or nucleus, and green punctate dots (PAX‐5) distinguished tumor cells from immune cells. PD‐L1 mRNA expression levels were categorized into 5 grades according to the manufacturer's scoring guideline: score 0, no staining or < 1 dot per 10 cells; score 1, 1‐3 dots per cell; score 2, 4‐9 dots per cell and no dot clusters; score 3, 10‐15 dots per cell and < 10% of positive cells contain dot clusters; score 4, >15 dots per cell and > 10% of positive cells contain dot clusters. Scores of 0, 1, and 2 were classified as negative, and 3 and 4 were positive.

### 2.5 Statistical analysis

The interobserver concordance and concordance between the results of PD‐L1 presence detected by IHC, RNAscope, and FISH was evaluated by the Kappa value. A Kappa value ≤ 0.40 was poor concordance; 0.41‐0.75 was moderate concordance; >0.75 was strong concordance. The relationship between PD‐L1 alterations and clinicopathological characteristics of the patients was compared using the chi‐squared test or Fisher's exact test. The treatment outcomes were measured by overall survival (OS), which was defined as the time from the day of diagnosis to the day of death or the day of the last follow‐up. Estimates of OS were calculated by the Kaplan‐Meier method, and the log‐rank test was used to compare the difference between the two groups. Cox regression models were used to estimate the prognostic risk factors. All *P*‐values involved in this study are 2 sided, and a *P*‐value < 0.05 was considered significant. All statistical analyses were performed with the SPSS 20.0 software.

## 3 RESULTS

### 3.1 PD‐L1 IHC staining analysis and cutoff value determination

The 126 patients included 66 males and 60 females with ages ranging from 15 to 89 years (median 59 years). According to the 2017 WHO classification, 94 of cases were reclassified into DLBCL‐NOS, 25 were PMBCL and 7 were DHL. All the 126 patients had available samples and presented optimal results for IHC, with SP263 100% (126/126) and SP142 100% (126/126).

PD‐L1 expression assessed by SP263 showed generally stronger and more complete membrane staining (Figure 1A and B); while in most cases, the PD‐L1 signal shown by SP142 appeared weak, dim, and discontinuous (Figure 1D and E). In addition, SP263 had an extremely clean background, while the background of SP142 staining was slightly inferior to SP263 (Figure 1C and F). Resultantly, for the SP263 staining, TC and IC could be identified more easily by the morphological features of the cells (Figure 1A and B), while for SP142 staining, B‐cell‐specific biomarker could help the clear discrimination of TC from IC (Figure 1D and E).

The percentage of TC, IC, and combined cell positive staining by SP263 and SP142 for each case was estimated. The comparison between the two antibodies in TC, IC, and combined cell staining is shown in Figure 1. SP263‐ and SP142‐positive cells showed a similar linear distribution trend, and SP263 could recognize more PD‐L1‐positive cells (Figure 1G‐I), especially in IC (Figure 1H).

To determine the potential predicting ability, a series of cutoffs with 5%, 10%, 15%, 20%, 25%, 30%, 40%, or 50% for TC, IC, and combined PD‐L1‐positive for SP263 and SP142 were analyzed with the corresponding survival of patients with DLBCL‐NOS (Table 1). For SP263, greater than 25% for IC and combined PD‐L1‐positive showed association with shorter OS, while all cutoffs for TC did not. For SP142, cutoff greater than 10% PD‐L1 expression for SP142 TC all showed significant *P* value; and greater than 20% PD‐L1‐positive for IC and 15% for combined all was associated with shorter OS. Therefore, in DLBCL‐NOS, the potential cutoff of SP263 could be 25%, while for SP142 10% TC and 20% for both IC and combined could be reasonable. Despite more positive stained cells in SP263, while considering the clinical significance, both of results from SP263 and SP142 were compatible, and SP142 was even more predictable in prognosis than SP263. In addition, combined SP263 and SP142 might be more suitable for practical prediction, since combined PD‐L1 expression was much easier for staining evaluation and had the comparable prognostic value with TC and IC. Besides, based on the above potential cutoff, scoring of PD‐L1‐positive TC, IC, and combined cell in three categories showed moderate to strong interobserver concordance coefficients (*k* = 0.462‐1.000, Table S1), and interobserver concordance in combined cells was higher than that in TC and IC for both SP263 and SP142 (Table S1).

**Table 1:** The relationship between PD‐L1 positive by IHC with different cutoff and corresponding survival significance in DLBCL‐NOS

SP263	Cutoff (%)	n (%)	*P*	SP142	Cutoff (%)	n (%)	*P*
TC	5	75 (79.8)	0.297	TC	5	58 (61.7)	0.178
	10	39 (41.5)	0.241		10	26 (27.7)	0.005
	15	29 (30.9)	0.281		15	21 (22.3)	0.008
	20	26 (27.7)	0.098		20	19 (20.2)	0.002
	25	18 (19.1)	0.107		25	13 (13.8)	0.046
	30	17 (18.1)	0.052		30	13 (13.8)	0.046
	40	11 (11.7)	0.069		40	8 (8.5)	0.015
	50	10 (10.6)	0.063		50	6 (6.4)	0.001
IC	5	90 (95.7)	0.965	IC	5	87 (92.6)	0.600
	10	61 (64.9)	0.187		10	46 (48.9)	0.150
	15	22 (23.4)	0.088		15	18 (19.1)	0.133
	20	20 (21.3)	0.105		20	13 (13.8)	0.013
	25	15 (16.0)	0.006		25	9 (9.6)	0.001
	30	15 (16.0)	0.006		30	9 (9.6)	0.001
	40	8 (8.5)	0.001		40	6 (6.4)	0.002
	50	6 (6.4)	0.001		50	3 (3.2)	0.010
Combined	5	92 (97.9)	0.371	Combined	5	88 (93.6)	0.625
	10	66 (70.2)	0.249		10	51 (54.3)	0.067
	15	38 (40.4)	0.487		15	29 (30.9)	0.036
	20	35 (37.2)	0.243		20	23 (24.5)	0.002
	25	23 (24.5)	0.028		25	17 (18.1)	<0.001
	30	22 (24.5)	0.013		30	14 (14.9)	<0.001
	40	12 (12.8)	0.002		40	10 (10.6)	<0.001
	50	11 (11.7)	0.010		50	6 (6.4)	0.001

Abbreviations: DLBCL‐NOS, diffuse large B‐cell lymphoma, not otherwise specified; IC, immune cell; IHC, immunohistochemistry; PD‐L1, programmed cell death ligand 1; TC, tumor cell.

### 3.2 PD‐L1 protein expression and pathological features

The results of PD‐L1 expression status in various sets of categories and the pathological features were summarized (Table 2). In all of 126 cases, the prevalence rates of combined^+^, TC^+^, and IC^+^ by SP263 were 33.3%, 27.8%, and 21.4%, respectively, and the prevalence rates by SP142 were 34.9%, 39.7%, and 19.8%. In 94 cases of DLBCL‐NOS, the prevalence rates of combined^+^, TC^+^, and IC^+^ by SP263 were 24.5%, 19.1%, and 16.0%, respectively, and the prevalence rates by SP142 were 24.5%, 27.7%, and 13.8%, respectively (Table 2). The similar cutoff determination analysis was also performed in patients with PMBCL and DHL, but none of the cutoff value showed prognostic significance (data not shown), so the same cutoff was set in PMBCL and DHL. In 25 cases of PMBCL, the prevalence rates of combined^+^, TC^+^, and IC^+^ by SP263 were 56.0%, 56.0%, and 36.0%, respectively, and the prevalence rates by SP142 were 64.0%, 76.0%, and 32.0%, respectively. In 7 cases of DHL, the prevalence rates of combined^+^, TC^+^, and IC^+^ by SP263 were 71.4%, 42.9%, and 42.9%, respectively, and the prevalence rates by SP142 were 71.4%, 71.4%, and 57.1%, respectively. The frequency of PD‐L1 TC expression by both SP263 and SP142 in PMBCL and DHL was significantly higher than that in DLBCL‐NOS (*P* = 0.001 and *P* < 0.001, Table 2). The results of SP263 and SP142 showed moderate to strong concordance in all of categories (*k* = 0.462‐1.000, Table S2). The combined^+^DLBCL detection by SP263 and SP142 was consistent in 90.5% (114/126) cases, including 37 cases with positive expression and 77 cases with negative expression (*k* = 0.788; Table S2).

**Table 2:** PD‐L1 expression status and pathological features of 126 patients

	SP263 (126)			SP142 (126)			RNAscope (52)			FISH (119)		
	TC +	TC −	*P*	TC +	TC −	*P*	+	−	*P*	Amplification & gain	Normal locus	*P*
	n (%)	n (%)		n (%)	n (%)		n (%)	n (%)		n (%)	n (%)	
All cases	35 (27.8)	91 (72.2)		50 (39.7)	76 (60.3)		19 (36.5)	33 (63.5)		32 (26.9)	87 (73.1)	
DLBCL‐NOS	18 (19.1)	76 (80.9)	0.001	26 (27.7)	68 (72.3)	<0.001	5 (17.2)	24 (82.8)	0.005	11 (12.0)	81 (88.0)	<0.001
PMBCL	14 (56.0)	11 (44.0)		19 (76.0)	6 (24.0)		13 (61.9)	8 (38.1)		19 (82.6)	4 (17.4)	
DHL	3 (42.9)	4 (57.1)		5 (71.4)	2 (28.6)		1 (50.0)	1 (50.0)		2 (50.0)	2 (50.0)	
DLBCL‐NOS												
GCB	2 (6.5)	29 (93.5)	0.029*	7 (22.6)	24 (77.4)	0.440	3 (30.0)	7 (70.0)	0.306*	1 (3.3)	29 (96.7)	0.095*
Non‐GCB	16 (25.4)	47 (74.6)		19 (30.2)	44 (69.8)		2 (10.5)	17 (89.5)		10 (16.1)	52 (83.9)	
DEL	5 (18.5)	22 (81.5)	0.921	7 (25.9)	20 (74.1)	0.811	0 (0)	4 (100.0)	<0.999*	3 (11.5)	23 (88.5)	<0.999*
non‐DEL	13 (19.4)	54 (80.6)		19 (28.4)	48 (71.6)		5 (20.0)	20 (80.0)		8 (12.1)	58 (87.9)	
IB	6 (50.0)	6 (50.0)	0.004	6 (50.0)	6 (50.0)	0.064	1 (50.0)	1 (50.0)	0.320*	3 (25.0)	9 (75.0)	0.153*
CB	12 (14.6)	70 (85.4)		20 (24.4)	62 (75.6)		4 (14.8)	23 (85.2)		8 (10.0)	72 (90.0)	

Abbreviations: CB, centroblastic variant; DEL, double‐expressor lymphoma; DHL, double‐hit lymphoma; DLBCL‐NOS, diffuse large B‐cell lymphoma, not otherwise specified; FISH, fluorescence in situ hybridization; GCB, germinal center B‐cell‐like; IB, immunoblastic variant; IC, immune cell; PD‐L1, programmed cell death ligand 1; PMBCL, primary mediastinal large B‐cell lymphoma; TC, tumor cell.

* Fisher's exact test.

Among patients with DLBCL‐NOS, 31 (33.0%) cases were diagnosed as GCB type DLBCL, and 63 (67.0%) cases were non‐GCB type DLBCL. DEL was diagnosed in 27 (28.7%) cases. As to histologic classification, 12 (12.8%) cases were grouped as immunoblastic variant subtype, and 82 (87.2%) cases were centroblastic variant subtype. TC^+^ by SP263 was more frequently occurred in non‐GCB type DLBCL‐NOS and IB type DLBCL‐NOS (*P* = 0.029 and *P* = 0.004, Table 2), while SP142 expression showed no difference in COO and histological subtype (*P* = 0.440 and *P* = 0.064, Table 2). PD‐L1 protein expression showed no statistical difference in DEL and non‐DEL.

### 3.3 Clinical features and prognostic analysis of patients with PD‐L1 expression by IHC

A total of 117 patients had available and complete clinical information, of which 91 patients were DLBCL‐NOS, 19 cases were PMBCL and 7 cases were DHL. The clinical features of 91 DLBCL‐NOS patients and the association with PD‐L1 alterations are summarized (Table 3). Among 91 of patients with DLBCL‐NOS, 65.9% of them accepted R‐CHOP/R‐CHOP‐like therapy; 8.8% accepted other chemotherapy, 17.6% were resected only, and 7.7% with no therapy. About 15.4% (14/91) patients accepted autologous stem cell transplantation. Positive combined PD‐L1 (SP263 and SP142) was more frequently found in patients with more than one extranodal site involved (*P* = 0.006, *P* = 0.042, respectively), higher ECOG PS scores (*P* = 0.001, *P* < 0.001, respectively), high IPI risk (*P* = 0.012, *P* = 0.005, respectively), and poor treatment response (*P* = 0.095, *P* = 0.002, respectively). Besides, patients with positive SP263 expression were more likely to relapse (*P* = 0.029). Owing to the small size of the cohort, patients with PMBCL and DHL showed no relationship with clinical features (data not shown).

**Table 3:** Relationship between clinical characteristic of patients and PD‐L1 status in DLBCL‐NOS

	Total	Combined SP263			Combined SP142			RNAscope			FISH		
		+	−	*P*	+	−	*P*	+	−	*P*	Amplification & gain	Normal locus	*P*
	n (%)	n (%)	n (%)		n (%)	n (%)		n (%)	n (%)		n (%)	n (%)	
	n = 91		n = 91			n = 91			n = 27			n = 89	
Age													
Median	66	69	65		70	65		64	59		66	66	
(Range)	(19‐86)	(52‐80)	(19‐86)		(52‐80)	(19‐86)		(57‐73)	(28‐86)		(28‐84)	(19‐86)	
>60	54 (59.3)	14 (63.6)	40 (58.0)	0.638	13 (61.9)	41 (58.6)	0.785	2 (50.0)	10 (43.5)	<0.999*	7 (63.6)	46 (59.0)	<0.999*
Sex													
Male	47 (51.6)	11 (50.0)	36 (52.2)	0.859	11 (52.4)	36 (51.4)	0.939	1 (25.0)	6 (26.1)	<0.999*	5 (45.5)	42 (53.8)	0.602
Female	44 (48.4)	11 (50.0)	33 (47.8)		10 (47.6)	34 (48.6)		3 (75.0)	17 (73.9)		6 (54.5)	36 (46.2)	
Ann Arbor stage > 2	69 (75.8)	18 (81.8)	51 (73.9)	0.573*	19 (90.5)	50 (71.4)	0.088*	3 (75.0)	18 (78.3)	<0.999*	11 (100.0)	57 (73.1)	0.060*
Extranodal sites > 1	43 (47.3)	16 (72.7)	27 (39.1)	0.006	14 (66.7)	29 (41.4)	0.042	3 (75.0)	15 (65.2)	<0.999*	9 (81.8)	32 (41.0)	0.020*
Elevated serum LDH	33 (40.2)	10 (58.8)	23 (35.4)	0.079	10 (58.8)	23 (35.4)	0.079	3 (75.0)	9 (39.1)	0.294*	4 (44.4)	29 (40.8)	<0.999*
ECOG PS > 1	24 (26.4)	12 (54.5)	12 (17.4)	0.001	12 (57.1)	12 (17.1)	<0.001	1 (25.0)	6 (26.1)	<0.999*	5 (545.5)	19 (24.4)	0.140
IPI risk > 2	45 (49.5)	16 (72.7)	29 (42.0)	0.012	16 (76.2)	29 (41.4)	0.005	3 (75.0)	13 (56.5)	0.624*	8 (72.7)	37 (47.4)	0.197*
B symptoms	34 (37.4)	8 (36.4)	26 (37.7)	0.911	11 (52.4)	23 (32.9)	0.105	2 (50.0)	15 (65.2)	0.613*	6 (54.5)	27 (34.6)	0.200
Treatment													
R‐CHOP/R‐CHOP‐like	60 (65.9)	13 (59.1)	47 (68.1)		11 (52.4)	49 (70.0)		3 (75.0)	17 (73.9)		6 (54.5)	53 (66.7)	
Other chemotherapy	8 (8.8)	0 (0)	8 (11.6)		0 (0)	8 (11.4)		0 (0)	0 (0)		1 (9.1)	7 (9.0)	
Resection only	16 (17.6)	5 (22.7)	11 (15.9)		7 (33.3)	9 (12.9)		1 (25.0)	6 (26.1)		2 (18.2)	14 (17.9)	
No therapy	7 (7.7)	4 (18.2)	3 (4.3)		3 (14.3)	4 (5.7)		0 (0)	0 (0)		2 (18.2)	5 (6.4)	
ASCT (total)	14 (15.4)	2 (9.1)	12 (17.4)	0.504*	2 (9.5)	12 (17.1)	0.508*	0 (0)	6 (26.1)	0.545*	0 (0)	13 (16.7)	0.356*
Treatment response													
CR	49 (53.8)	8 (40.0)	41 (63.1)	0.095	5 (26.3)	44 (66.7)	0.002	2 (50.0)	13 (59.1)	0.425	2 (22.2)	45 (60.8)	0.046
PR	26 (28.6)	10 (50.0)	16 (24.6)		12 (63.2)	14 (21.2)		2 (50.0)	5 (22.7)		6 (66.7)	20 (27.0)	
SD	10 (11.0)	2 (10.0)	8 (12.3)		2 (10.5)	8 (12.1)		0 (0)	4 (18.2)		1 (11.1)	9 (12.2)	
Not evaluable	6 (6.6)												
Relapse	16 (18.4)	7 (35.0)	9 (13.4)	0.029	5 (27.8)	11 (15.9)	0.248	2 (50.0)	4 (19.0)	0.234*	3 (30.0)	13 (17.3)	0.390*

Abbreviations: ASCT, autologous stem cell transplantation; CR, complete response/remission; DLBCL‐NOS, diffuse large B‐cell lymphoma, not otherwise specified; ECOG, Eastern Cooperative Oncology Group; FISH, fluorescence in situ hybridization; IPI, international prognostic index; LDH, lactate dehydrogenase; PD‐L1, programmed cell death ligand 1; PR, partial response/remission; PS, performance status; SD, stable disease.

* Fisher's exact test.

One hundred and ten patients including 16 of PMBCLs, 88 of DLBCL‐NOSs, and 6 of DHLs had follow‐up information with a median time of 33 months and ranging from 1 to 106 months. Sixteen patients were lost to follow‐up and handled as censored data. Overall, 28.4% (25/88) patients died due to DLBCL‐NOS progression; 18.8% (3/16) of patients died due to PMBCL; 66.7% (4/6) of patients died due to DHL; and 5 patients died for other reasons. About 18.2% (16/88) patients with DLBCL‐NOS experienced recurrence. The five‐year survival rate of patients with DLBCL‐NOS was 71.6%. In DLBCL‐NOS, the Kaplan‐Meier analysis showed that patients with combined^+^ DLBL‐NOS by IHC had inferior OS (Table 1, Figure 5A and B). Moreover, both for SP263 and SP142, with cutoff value increased, a more obvious or a resurgent relationship with prognosis was shown (Table 1), which suggested that patients with higher protein expression of PD‐L1 had a closer relationship with poor outcome. In the Cox regression models, the variables included were gender, age, IPI, B symptoms, and PD‐L1 biomarkers. The multivariate analysis indicated that IC^+^ DLBCL‐NOS by SP263 and SP142 was both independent prognostic factors for DLBCL‐NOS (*P* = 0.027 and *P* = 0.037, Table 4). Patients with PMBCL and DHL showed no relationship between PD‐L1 expression and short OS (data not shown).

**Table 4:** Prognostic factors of OS of patients with DLBCL‐NOS

	Univariate analysis		Age‐ and sex‐adjusted analysis		Multivariate analysis1	
	*P*	HR(95%CI)	*P*	HR(95%CI)	*P*	HR(95%CI)
Gender	0.362	0.689 (0.309‐1.535)				
Age	0.536	1.009 (0.980‐1.039)				
IPI	0.001	5.080 (2.018‐12.786)	<0.001	5.929 (2.295‐15.319)	0.002	4.867 (1.810‐13.090)
B symptoms	0.064	2.103 (0.958‐4.619)	0.042	2.305 (1.030‐5.158)	0.240	1.687 (0.706‐4.034)
Combined SP263	0.035	2.415 (1.063‐5.485)	0.049	2.293 (1.005‐5.228)	0.115	2.018 (0.842‐4.837)
TC SP263	0.119	0.172 (0.017‐1.751)	0.156	1.988 (0.770‐5.136)	0.453	1.467 (0.539‐3.990)
IC SP263	0.011	3.165 (1.309‐7.650)	0.008	3.462 (1.390‐8.623)	0.027	2.912 (1.133‐7.487)
Combined SP142	0.004	3.395 (1.471‐7.837)	0.006	3.224 (1.389‐7.485)	0.051	2.407 (0.997‐5.809)
TC SP142	0.008	2.988 (1.327‐6.729)	0.009	2.940 (1.301‐6.641)	0.074	2.162 (0.929‐5.031)
IC 142	0.021	3.282 (1.200‐8.973)	0.018	3.347 (1.228‐9.126)	0.037	3.046 (1.069‐8.680)
RNAscope	0.780	1.360 (0.158‐11.717)	0.775	1.375 (0.156‐12.151)	0.171	7.928 (0.410‐153.302)
Amplification and gain	0.017	3.105 (1.227‐7.855)	0.011	3.398 (1.328‐8.696)	0.080	2.360 (0.903‐6.171)

Abbreviations: DLBCL‐NOS, diffuse large B‐cell lymphoma, not otherwise specified; IC, immune cell; IPI, international prognostic index; OS, overall survival; TC, tumor cell.

### 3.4 9p24.1 locus alterations and corresponding prognostic analysis

**Figure 2:**
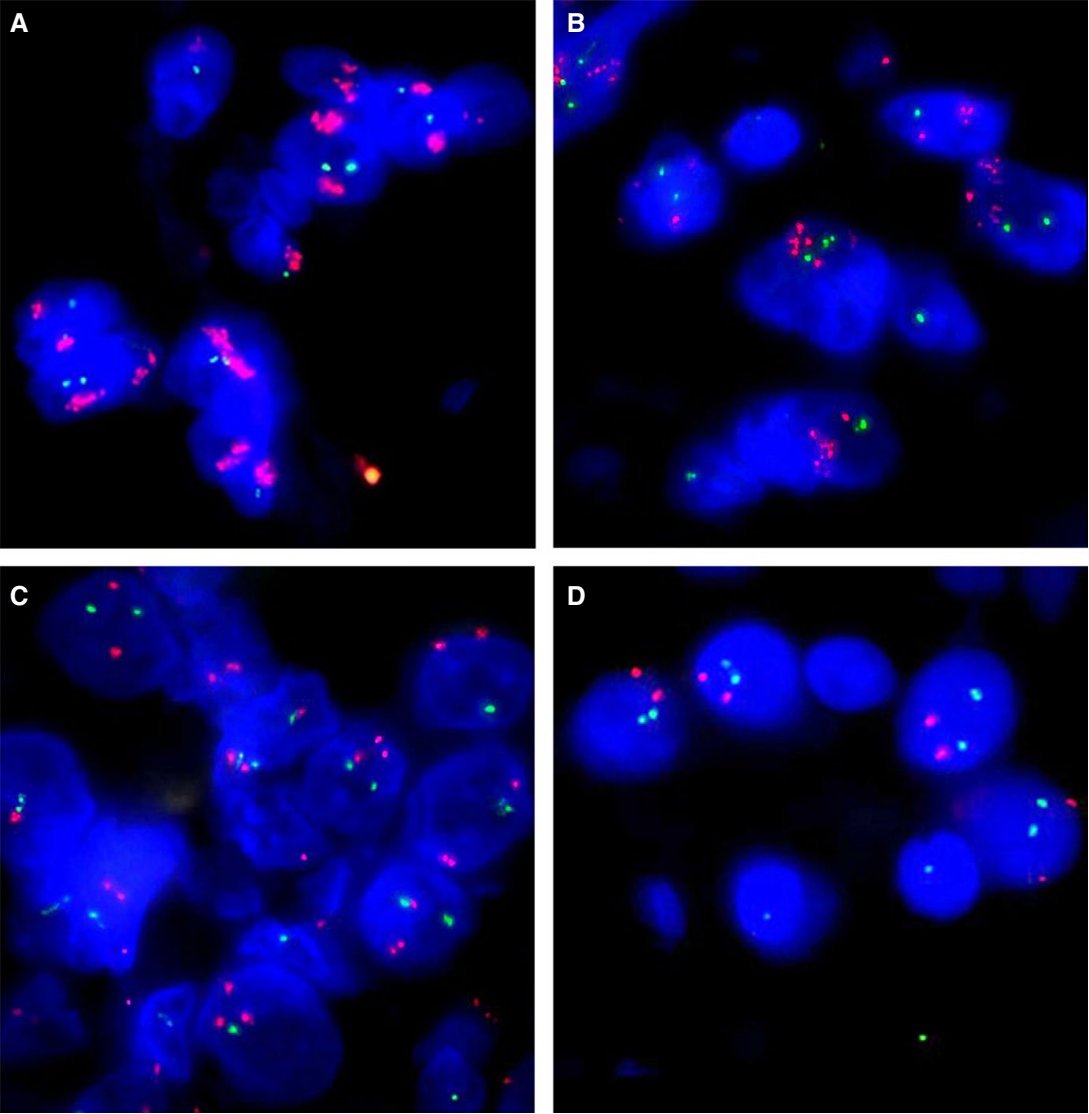
Cytogenetic changes affected the programmed cell death ligand‐1 (PD‐L1) locus detected by fluorescence in situ hybridization (FISH) in DLBCL. As shown, the presence of 5 or more red signals targeting PD‐L1 was determined as amplification (A, B), whereas three or four red signals were classified as gain (C). Normal cases were defined when two red signals targeting PD‐L1, and two green signals targeting ABL1 were present in one nucleus (D)

**Figure 3:**
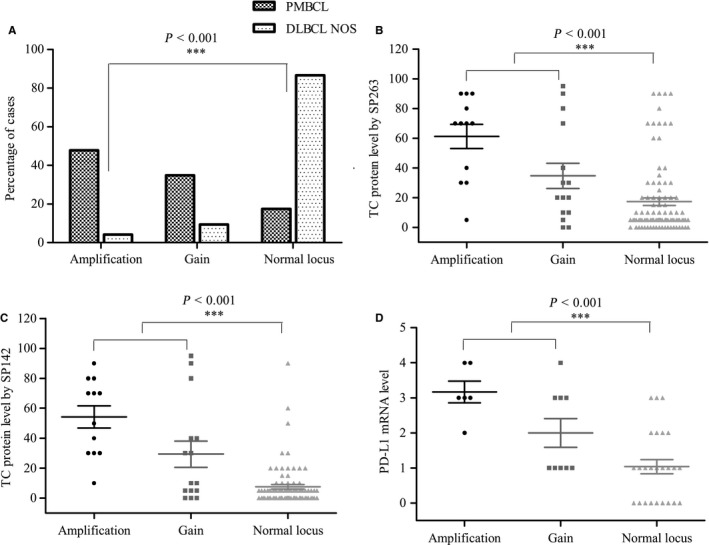
The distribution of 9P24.1 locus alterations. A Patients with primary mediastinal B‐cell lymphoma (PMBCL) were more frequently affected by 9p24.1 locus alterations than patients with diffuse large B‐cell lymphoma, not otherwise specified (DLBCL‐NOS); B‐D In all the cases, higher programmed cell death ligand‐1 (PD‐L1) expression at both the protein and mRNA levels was seen in patients with 9p24.1 locus alterations, especially in 9p24.1 amplification

FISH was performed on all 126 samples, and 119 samples yielded usable results. Among them, 15 cases were identified with 9p24.1 locus amplifications (12.6%) and 17 cases with gains (14.3%) (Figure 2A‐D). The alterations of the 9p24.1 locus were far more frequently occurred in PMBCL (Figure 3A) and DHL than in DLBCL‐NOS (*P* < 0.001, Table 2). In patients with PMBCL, the positive rates for amplification and gain were 47.8% (11/23) and 34.8% (8/23), and for DHL, the responding rates were 0% (0/4) and 50% (2/4). While for DLBCL‐NOS, 4.3% (4/92) of cases showed amplification and 7.6% (7/92) cases showed gains. The 9p24.1 locus alterations were significantly associated with TC PD‐L1 expression, especially in PMBCL (Table S3). And an obvious increased level of PD‐L1 protein and mRNA expression was presented in 9p24.1 amplification (*P* < 0.001, *P* < 0.001, *P* < 0.001, respectively; Figure 3B‐D).

In DLBCL‐NOS, Patients with 9p24.1 locus alterations were associated with more than one extranodal site involved (*P* = 0.020, Table 3) and poor treatment response (*P* = 0.046, Table 3). Patients with 9p24.1 locus amplification and gain were associated with shorter OS in DLBCL‐NOS (*P* = 0.004, Figure 5C). However, patients with PMBCL and DHL showed no relationship with clinical features and prognosis (data not shown).

### 3.5 PD‐L1 expression by RNAscope

**Figure 4:**
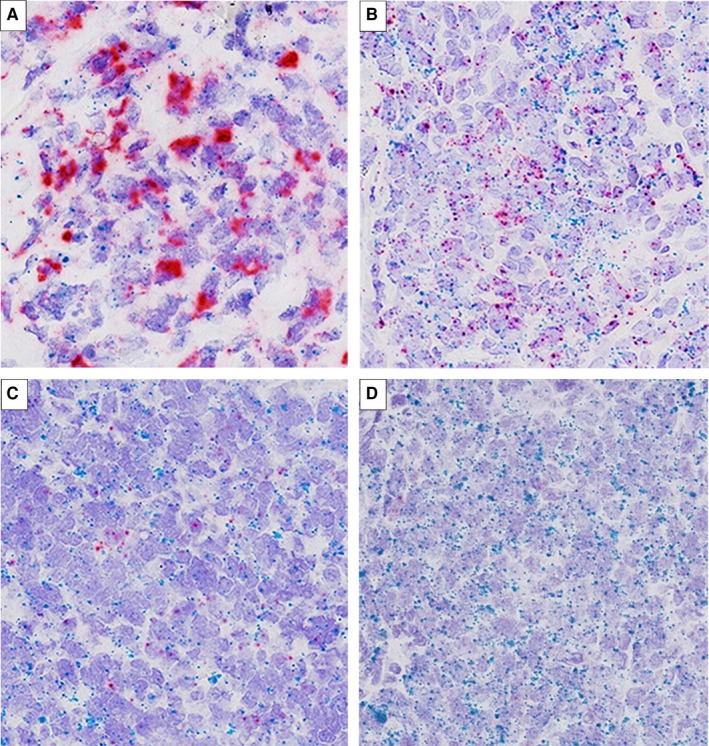
PD‐L1 mRNA expression by RNAscope (×400). Positive PD‐L1 expression (score = 3‐4) showed the dot clusters of PD‐L1 (red) staining (A, B). The negative cases showed fewer red signals (score = 0‐2) (C, D)

**Figure 5:**
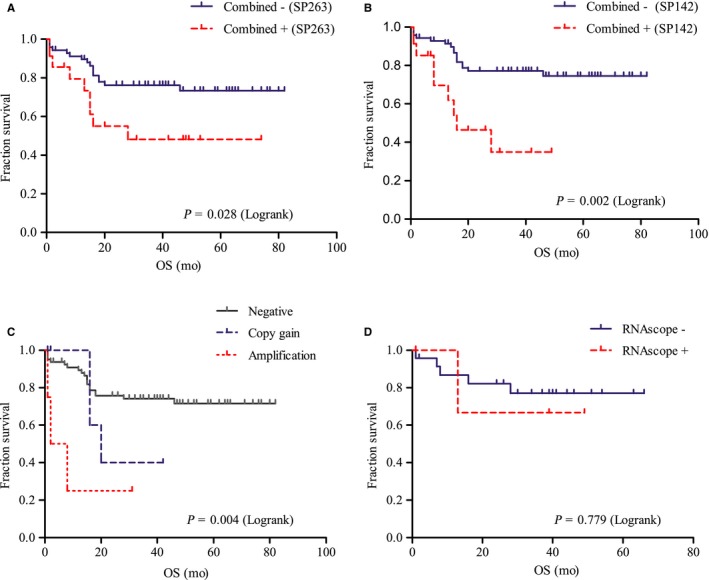
The relationship between programmed cell death ligand‐1 (PD‐L1) status and overall survival (OS) in patients with diffuse large B‐cell lymphoma, not otherwise specified (DLBCL‐NOS). (A, B) Patients with combined^+^ DLBCL‐NOS by SP263 and SP142 had poor prognosis; (C) 9p24.1 locus alterations were associated with shorter OS; (D) There were no significant differences in OS between the different results of PD‐L1 mRNA expression in DLBCL‐NOS

RNAscope was performed on 58 cases of DLBCL, and 89.7% (52/58) of the results were available, including 29 cases of DLBCL‐NOS, 21 cases of PMBCL, and 2 cases of DHL. High PD‐L1 expression (score = 4) was presented in 5 (9.6%) cases; score 3 was presented in 14 (26.9%) cases; score 2 was presented in 7 (13.5%) cases; score 1 was presented in 16 (30.8%) cases; and score 0 was presented in 10 (19.2%) cases. Positive PD‐L1 mRNA overexpression (score = 3‐ 4) was detected in 36.5% (19/52) cases (Figure 4). The positive rates in PMBCL and DHL were 61.9% (13/21) and 50% (1/2), which was obviously higher than that in DLBCL‐NOS with 17.2% (5/29) (*P* = 0.005, Table 2). RNAscope presented moderate to strong concordance with PD‐L1 protein expression and 9p24.1 amplification in almost all of categories (k = 0.394‐1.000; Table S4). There was no relationship either between PD‐L1 detected by RNAscope and pathologicoclinical features (Table 3), or between PD‐L1 and prognosis (*P* = 0.779; Figure 5D).

## 4 DISCUSSION

In light of the successful application of anti‐PD‐1/PD‐L1 immunotherapy in solid tumors,^7^ clinical trials of anti‐PD‐1/PD‐L1 agents have been carried out in patients with DLBCL,^13^ and accurate evaluation of PD‐L1 status has become a major challenge in DLBCL. The value of a predictive biomarker should depend on its technical feasibility, biological significance, and therapy response. Several studies found that the status of PD‐L1 expression could predict response to PD‐1/PD‐L1 blockade and overall survival of the patients. Since our study did not cover the response rate of immunotherapy, so the prognostic significance was set as a “reference standard” to evaluate the predictive value of three methods including IHC with SP263 and SP142, RNAscope and FISH. We found that IHC was a reliable and convenient method, in which SP263 was easier to analysis, while SP142 was more predictable.

Currently, several clonal antibodies have been developed and used in the evaluation of PD‐L1 expression. In solid tumors, the thresholds of SP263 for both TC and IC were 25%, while the threshold for SP142 was classified into several degrees composed of 1%, 5%, 10%, and 50% for TC and IC.^14^, ^21^ While in DLBCL, the consensual diagnostic suitable cutoff has not been approved yet. Kiyasu et al^16^ based on the staining distribution defined the threshold of PD‐L1 in DLBCL and set the TC^+^ DLBCL as 30% and IC^+^ DLBCL as 20% with the anti‐PD‐L1 monoclonal antibody, clone EPR1161. In our study, various grades of cutoff value for SP263 and SP142 ranging from 5% to 50% were put into the evaluation with survival analysis in DLBCL‐NOS (Table 1) and the results showed that 25% was the applicable cutoff for SP263, and for SP142, 10% TC, and 20% for both IC and combined might be suitable, all of which were similar to the previous reports.^14^, ^21^ Specifically, using this cutoff, PD‐L1 protein expression rates by SP263 and SP142 were comparable, and closely correlated with the results of RNAscope or FISH. Moreover, SP263 and SP142 were both prognostic factors in DLBCL‐NOS in the Cox regression models (Table 4). Previous studies showed that SP142 was a weaker antibody for PD‐L1 expression detection in non‐small‐cell lung cancer.^21^, ^25^, ^26^ Our study showed similar results in DLBCL. SP263 staining density was stronger than SP142 which meant SP263 was easier to evaluate, and SP263 could stain more PD‐L1‐positive cells (Figure 1G‐I). Besides, the interobserver concordance in SP263 combined cells was higher than that in SP142 (Table S1). Although SP142 seemed to be weaker than SP263 as described, but we found that SP142 had a closer relationship with shorter OS, both for TC, IC, and combined suggested better predictive value in SP142.

TPS was used as a classical method to identify patients who had TC^+^ tumor and likely to respond to immunotherapy. While it is not enough only with the measurement of TC, IC PD‐L1‐positive expression was also proved to be a necessary predictive marker and associated with poor prognosis in several types of solid tumors.^27^ Xu‐Monette et al^28^ performed immunophenotypic profiling using a MultiOmyx immunofluorescence platform in 405 patients, and their results showed the critical role of the immune microenvironment and PD‐1 expression on CD8^+^ T cell and PD‐L1 expression on IC including tumor‐infiltrating T cell and macrophages both indicated inferior survival. Our study showed similar prognostic role of IC, patients with IC^+^ DLBCL‐NOS by SP263 and SP142 also had worse prognosis and were independent risk factors for DLBCL‐NOS (Figure 5, Table 4), while those with TC^+^ DLBCL‐NOS by SP263 did not. In addition, we also calculated the combined PD‐L1 expression level with the modified CPS algorithm, which was approved by FDA on 22 Sept. 2017 for 22C3 antibody and was proved had better predictive ability than TPS in gastric cancer.^22^ In our study, combined PD‐L1 expression showed comparable prognostic significance with TC and IC for both SP263 and SP142 (Table 1); moreover, combined PD‐L1 staining was easier to evaluate than TC and IC, so combined PD‐L1 positive by IHC might be a better option and a reliable predictive marker in DLBCL.

The molecular genetic mechanism of overexpression of PD‐L1 is very complex, and PD‐L1 gene amplification, translocation, 3’‐UTR disruption, and the TME (tumor microenvironment) can all lead to upregulation of PD‐L1.^9^, ^24^, ^29^, ^30^, ^31^ Previous studies have shown that PD‐L1 locus amplification and gain were the main reason for PD‐L1 expression in PMBCL, and the positive rates were high with 55%‐65%, while for DLBCL was much lower with 15%.^17^, ^24^ Our study revealed similar results, and 9p24.1 amplification was closely associated with high PD‐L1 expression level (Figure 4), implying that a different mechanism is involved in the upregulation of PD‐L1 in PMBCL and DLBCL‐NOS, and 9p24.1 locus alteration was indeed the main reason for the high expression of PD‐L1 in PMBCL. Although our results showed 9p24.1 locus alteration was rare in DLBCL‐NOS, it still showed a close relationship with poor prognosis (Figure 5). Thus, PD‐L1 amplification and gain still had predictive significance in DLBCL‐NOS and can be auxiliary to other methods such as IHC.

PD‐L1 mRNA expression level has been measured in solid tumors, including gastric cancer and non‐small‐cell lung cancer by using RNA‐seq or ISH with RNAscope, and it was found to be highly consistent with their IHC detection results.^19^, ^20^, ^32^, ^33^ Benefiting from the unique “double‐Z” probes, independent of antibody, and visible results on tissue sections, the RNAscope method seemed to be more sensitive than IHC and more objective than RNA‐seq. In our study, the expression of PD‐L1 at the mRNA level was indeed significantly associated with expression of PD‐L1 at the protein level and PD‐L1 locus amplification, consistent with previous reports. While we found PD‐L1 expression by RNAscope had no relationship to the prognosis of patients with DLBCL‐NOS (Figure 5). This might be caused by the limited number of cases and the scoring method for the RNAscope assay, which was continuous and subjective. Sun et al^34^ also used RNAscope method to evaluate PD‐L1 mRNA expression in DLBCL, and the cutoff used was different from our study. We tried the cutoff reported and other possible cutoff value, but none of them showed prognostic value. Nevertheless, the high sensitivity and consistency with IHC and genetic changes indicated that RNAscope could be used as a supplementary assay, especially for analysis of PMBCL.

Previous study showed that in tumors with high PD‐L1 amplification occurrence and/or PD‐L1 overexpression, patients seemed to be more susceptible to PD‐1/PD‐L1 blockade,^17^, ^18^ In our study, both of PD‐L1 expression and genetic locus alterations were consistently higher in PMBCL, while PMBCL patients with PD‐L1 alterations showed no relationship with poor outcome. Unlike DLBCL‐NOS, most of PMBCL showed less heterogeneity and better prognoses. Hence, despite without clinical significance, the category of PMBCL itself is still an immunotherapy sensitive cancer, just like some other cancer types, such as Hodgkin's lymphoma, EBV‐positive type of gastric cancer might be sensitive to immunotherapy without needing biomarker test. In our study, PD‐L1 expression rate and 9p24.1 alterations were also higher in DHL, while case number was limited, larger cohort, and further study for PD‐L1 expression level and underlying mechanism in DEL were needed.

In conclusion, the detection of PD‐L1 by IHC could be a reliable and easy method in predicting the response to PD‐1/PD‐L1 blockade for DLBCL‐NOS, especially the combined PD‐L1 staining recording could be used in practice. The cutoff for SP142 and SP263 should be different. The staining of SP263 was easier to evaluate and was more sensitive, while SP142 was a more accurate biomarker for assessing the prognosis of patients with DLBCL‐NOS. FISH and RNAscope could also be considered as a supplementary diagnostic assay. In PMBCL, the high frequency of PD‐L1 locus amplification and PD‐L1 overexpression probably indicated their good response to immunotherapy. Nevertheless, a concrete cutoff of PD‐L1 expression and detected method chosen should be determined by their relation to the outcome or response to immunotherapy in clinical practice; hence, our study was more a suggestion than a principle in PD1/PD‐L1 blockade prediction, and treatment response and larger cohort were needed in the future study.

## CONFLICT OF INTEREST

No conflict of interest disclaimed.
