# Supplementary material for: Comparison of PD‐L1 detection assays and corresponding significance in evaluation of diffuse large B‐cell lymphoma
Source: Cancer Med. 2019 May 31;8(8):3831–45. doi: 10.1002/cam4.2316 (PMC6639200; doi:10.1002/cam4.2316)
Supplement: Supplementary file 1 [file CAM4-8-3831-s001.doc]

**SUPPLEMENTAL TABLE 1** Interobserver concordance analysis

| **Pathologist A** | | | | | | | | | | | | | | | | | | | | |
| --- | --- | --- | --- | --- | --- | --- | --- | --- | --- | --- | --- | --- | --- | --- | --- | --- | --- | --- | --- | --- |
|  |  |  | **SP263 combined** | | | **SP263 TC** | | | **SP263 IC** | | | **SP142 combined** | | | **SP142 TC** | | | **SP142 IC** | | |
|  |  |  | **+** | **-** | **k** | **+** | **-** | **k** | **+** | **-** | **k** | **+** | **-** | **k** | **+** | **-** | **k** | **+** | **-** | **k** |
|  |  |  | **n(%)** | **n(%)** |  | **n(%)** | **n(%)** |  | **n(%)** | **n(%)** |  | **n(%)** | **n(%)** |  | **n(%)** | **n(%)** |  | **n(%)** | **n(%)** |  |
|  | All cases | + | 42(97.7) | 1(2.3) | 0.982 | 32(88.9) | 4(11.1) | 0.863 | 22(68.8) | 10(31.2) | 0.650 | 42(93.3) | 3(6.7) | 0.913 | 45(90.0) | 5(10.0) | 0.883 | 23(82.1) | 5(17.9) | 0.751 |
|  |  | - | 0(0) | 83(100.0) |  | 3(3.3) | 87(96.7) |  | 6(6.4) | 88(93.6) |  | 2(2.5) | 79(97.5) |  | 2(2.6) | 74(97.4) |  | 6(6.1) | 92(93.9) |  |
|  | DLBCL-NOS | + | 23(95.8) | 1(4.2) | 0.972 | 16(88.9) | 2(11.1) | 0.863 | 12(70.6) | 5(29.4) | 0.669 | 22(91.7) | 2(8.3) | 0.915 | 24(92.3) | 2(7.7) | 0.894 | 11(78.6) | 3(21.4) | 0.715 |
| Pathologist B |  | - | 0(0) | 70100.0) |  | 2(2.6) | 74(97.4) |  | 4(5.2) | 73(94.8) |  | 1(1.4) | 69(98.6) |  | 2(2.9) | 66(97.1) |  | 4(5.0) | 76(95.0) |  |
|  | PMBCL | + | 14(100.0) | 0(0) | 1.000 | 13(92.9) | 1(7.1) | 0.838 | 7(70.0) | 3(30.0) | 0.576 | 16(94.1) | 1(5.9) | 0.911 | 17(89.5) | 2(10.5) | 0.803 | 8(80.0) | 2(20.0) | 0.746 |
|  |  | - | 0(0) | 11(100.0) |  | 1(9.1) | 10(90.9) |  | 2(13.3) | 13(86.7) |  | 0(0) | 8(100.0) |  | 0(0) | 6(100.0) |  | 1(6.7) | 14(93.3) |  |
|  | DHL | + | 5(100.0) | 0(0) | 1.000 | 3(75.0) | 1(25.0) | 0.720 | 3(60.0) | 2(40.0) | 0.462 | 4(100.0) | 0(0) | 0.696 | 4(80.0) | 1(20.0) | 0.696 | 4(100.0) | 0(0) | 0.696 |
|  |  | - | 0(0) | 2(100.0) |  | 0(0) | 3(100.0) |  | 0(0) | 2(100.0) |  | 1(33.3) | 2(66.7) |  | 0(0) | 2(100.0) |  | 1(33.3) | 2(66.7) |  |

DLBCL-NOS, diffuse large B-cell lymphoma, not otherwise specified; PMBCL, primary mediastinal large B-cell lymphoma; DHL, double hit lymphoma; TC, tumor cell; IC, immune cell.
