# Supplementary material for: Comparison of PD‐L1 detection assays and corresponding significance in evaluation of diffuse large B‐cell lymphoma
Source: Cancer Med. 2019 May 31;8(8):3831–45. doi: 10.1002/cam4.2316 (PMC6639200; doi:10.1002/cam4.2316)
Supplement: Supplementary file 2 [file CAM4-8-3831-s002.doc]

**SUPPLEMENTAL TABLE 2** Comparison of PD-L1 staining by SP263 and SP142

|  |  | **combined SP142** | | | | **TC SP142** | | | | **IC SP142** | | |
| --- | --- | --- | --- | --- | --- | --- | --- | --- | --- | --- | --- | --- |
|  |  | **+** | **-** | **k** | **+** | | **-** | **k** | **+** | | **-** | **k** |
|  |  | **n(%)** | **n(%)** |  | **n(%)** | | **n(%)** |  | **n(%)** | | **n(%)** |  |
| All cases | SP263 + | 37(88.1) | 5(11.9) | 0.788 | 33(94.3) | | 2(5.7) | 0.668 | 21(77.8) | | 6(22.2) | 0.758 |
|  | SP263 - | 7(8.3) | 77(91.7) |  | 17(18.7) | | 74(81.3) |  | 4(4.0) | | 95(96.0) |  |
| DLBCL-NOS | SP263 + | 18(78.3) | 5(21.7) | 0.712 | 16(88.9) | | 2(11.1) | 0.648 | 11(73.3) | | 4(26.7) | 0.748 |
|  | SP263 - | 5(7.0) | 66(93.0) |  | 10(13.2) | | 66(86.8) |  | 2(2.5) | | 77(97.5) |  |
| PMBCL | SP263 + | 14(100.0) | 0(0) | 0.834 | 14(100.0) | | 0(0) | 0.573 | 7(77.8) | | 2(22.2) | 0.733 |
|  | SP263 - | 2(18.2) | 9(81.8) |  | 5(45.5) | | 6(54.5) |  | 1(6.2) | | 15(93.8) |  |
| DHL | 5(100.0) | 0(0) |  | 1.000 | 3(100.0) | | 0(0) | 0.462 | 3(100.0) | | 0(0) | 0.720 |
|  | 0(0) | 2(100.0) |  |  | 2(50.0) | | 2(50.0) |  | 1(25.0) | | 3(75.0) |  |

PD-L1: programmed cell death ligand 1; DLBCL-NOS, diffuse large B-cell lymphoma, not otherwise specified; PMBCL, primary mediastinal large B-cell lymphoma; DHL, double hit lymphoma; TC, tumor cell; IC, immune cell.
