# Supplementary material for: Comparison of PD‐L1 detection assays and corresponding significance in evaluation of diffuse large B‐cell lymphoma
Source: Cancer Med. 2019 May 31;8(8):3831–45. doi: 10.1002/cam4.2316 (PMC6639200; doi:10.1002/cam4.2316)
Supplement: Supplementary file 3 [file CAM4-8-3831-s003.doc]

**SUPPLEMENTAL TABLE 3** The comparison of PD-L1 protein expression and PD-L1 locus alteration

|  |  | **SP263** | | | **SP142** | | |
| --- | --- | --- | --- | --- | --- | --- | --- |
|  |  | **TC +** | **TC -** | **k** | **TC +** | **TC -** | **k** |
|  |  | **n(%)** | **n(%)** |  | **n(%)** | **n(%)** |  |
| All cases | Amplification | 14(93.3) | 1(6.7) | 0.632 | 15(100.0) | 0(0) | 0.480 |
|  | Normal locus | 11(12.6) | 76(87.4) |  | 21(24.1) | 66(75.9) |  |
| DLBCL-NOS | Amplification | 4(100.0) | 0(0) | 0.401 | 4(100.0) | 0(0) | 0.248 |
|  | Normal locus | 10(12.3) | 71(87.7) |  | 18(22.2) | 63(77.8) |  |
| PMBCL | Amplification | 10(90.9) | 1(9.1) | 0.659 | 11(100.0) | 0(0) | 0.595 |
|  | Normal locus | 1(25.0) | 3(75.0) |  | 2(50.0) | 2(50.0) |  |
| DHL | Amplification | 0(0) | 2(100.0) | - | 1(50.0) | 1(50.0) | <0.001 |
|  | Normal locus | 0(0) | 2(100.0) |  | 1(50.0) | 1(50.0) |  |

PD-L1: programmed cell death ligand 1; DLBCL-NOS, diffuse large B-cell lymphoma, not otherwise specified; PMBCL, primary mediastinal large B-cell lymphoma; DHL, double hit lymphoma; TC, tumor cell.
