# Supplementary material for: Comparison of PD‐L1 detection assays and corresponding significance in evaluation of diffuse large B‐cell lymphoma
Source: Cancer Med. 2019 May 31;8(8):3831–45. doi: 10.1002/cam4.2316 (PMC6639200; doi:10.1002/cam4.2316)
Supplement: Supplementary file 4 [file CAM4-8-3831-s004.doc]

**SUPPLEMENTAL TABLE 4** The comparison of PD-L1 mRNA expression and PD-L1 protein expression, PD-L1 locus alteration

|  |  | **combined SP263** | | | **combined SP142** | | | **FISH** | | |
| --- | --- | --- | --- | --- | --- | --- | --- | --- | --- | --- |
|  |  | **+** | **-** | **k** | **+** | **-** | **k** | Amplification | Normal locus | **k** |
|  |  | **n(%)** | **n(%)** |  | **n(%)** | **n(%)** |  | **n(%)** | **n(%)** |  |
| All cases | RNAscope + | 15(78.9) | 4(21.1) | 0.706 | 15(78.9) | 4(21.1) | 0.559 | 8(66.7) | 4(33.3) | 0.670 |
|  | RNAscope - | 3(9.1) | 30(90.9) |  | 7(21.2) | 26(78.8) |  | 1(4.0) | 24(96.0) |  |
| DLBCL-NOS | RNAscope + | 4(80.0) | 1(20.0) | 0.758 | 4(80.0) | 1(20.0) | 0.512 | 0(0) | 4(100.0) | - |
|  | RNAscope - | 1(4.2) | 23(95.8) |  | 4(16.7) | 20(83.3) |  | 0(0) | 20(100.0) |  |
| PMBCL | RNAscope + | 10(76.9) | 3(23.1) | 0.507 | 10(76.9) | 3(23.1) | 0.394 | 8(8100.0) | 0(0) | 0.831 |
|  | RNAscope - | 2(25.0) | 6(75.0) |  | 3(37.5) | 5(62.5) |  | 1(20.0) | 4(80.0) |  |
| DHL | RNAscope + | 1(100.0) | 0(0) | 1.000 | 1(100.0) | 0(0) | 1.000 | 0(0) | 0(0) | - |
|  | RNAscope - | 0(0) | 1(100.0) |  | 0(0) | 1(100.0) |  | 0(0) | 0(0) |  |

PD-L1: programmed cell death ligand 1; IHC, immunohistochemistry; DLBCL-NOS, diffuse large B-cell lymphoma, not otherwise specified; PMBCL, primary mediastinal large B-cell lymphoma.
